# Low Doses of Bisphenol A Promote Human Seminoma Cell Proliferation by Activating PKA and PKG via a Membrane G-Protein–Coupled Estrogen Receptor

**DOI:** 10.1289/ehp.0800367

**Published:** 2009-02-11

**Authors:** Adil Bouskine, Marielle Nebout, Françoise Brücker-Davis, Mohamed Benahmed, Patrick Fenichel

**Affiliations:** 1 Institut National de la recherché Médicale (INSERM) U895, Team 5—Environment and Reproduction: Genomic and Nongenomic Mechanisms, University of Nice-Sophia-Antipolis, Faculty of Medicine, Nice, France; 2 Department of Reproductive Endocrinology, University Hospital of Nice, Nice, France

**Keywords:** bisphenol A, ER, estrogen receptor, GPCR, PKA, PKG, testicular germ cell cancer

## Abstract

**Background:**

Fetal exposure to environmental estrogens may contribute to hypofertility and/or to testicular germ cell cancer. However, many of these xenoestrogens have only a weak affinity for the classical estrogen receptors (ERs,) which is 1,000-fold less potent than the affinity of 17β-estradiol (E_2_). Thus, several mechanisms have been suggested to explain how they could affect male germ cell proliferation at low environmental relevant concentrations.

**Objectives:**

In this study we aimed to explore the possible promoting effect of bisphenol A (BPA) on human testicular seminoma cells. BPA is a well-recognized estrogenic endocrine disruptor used as a monomer to manufacture poly carbonate plastic and released from resin-lined food or beverage cans or from dental sealants.

**Methods and results:**

BPA at very low concentrations (10^−9^ to 10^−12^ M) similar to those found in human fluids stimulated JKT-1 cell proliferation *in vitro*. BPA activated both cAMP-dependent protein kinase and cGMP-dependent protein kinase pathways and triggered a rapid (15 min) phosphorylation of the transcription factor cAMP response-element–binding protein (CREB) and the cell cycle regulator retinoblastoma protein (Rb). This nongenomic activation did not involve classical ERs because it could not be reversed by ICI 182780 (an ER antagonist) or reproduced either by E_2_ or by diethylstilbestrol (a potent synthetic estrogen), which instead triggered a suppressive effect. This activation was reproduced only by E_2_ coupled to bovine serum albumin (BSA), which is unable to enter the cell. As with E_2_-BSA, BPA promoted JKT-1 cell proliferation through a G-protein–coupled nonclassical membrane ER (GPCR) involving a Gα_s_ and a Gα_i_/Gα_q_ subunit, as shown by the reversible effect observed by the corresponding inhibitors NF449 and pertussis toxin.

**Conclusion:**

This GPCR-mediated nongenomic action represents—in addition to the classical ER-mediated effect—a new basis for evaluating xenoestrogens such as BPA that, at low doses and with a high affinity for this GPCR, could interfere with the developmental programming of fetal germ cell proliferation and/or differentiation when they cross the placenta.

Endocrine-disrupting chemicals (EDCs) are hormone-like agents present in the environment that may alter the endocrine system of wildlife and humans. In particular, xenoestrogens have been hypothesized to be involved in developmental, reproductive, and malignant diseases by mimicking the natural hormone 17β-estradiol (E_2_) and interfering with endogenous endocrine regulation at specific periods, such as during fetal growth. Several organochloride pesticides—polychlorinated biphenyls (PCBs), phthalates, and bisphenol A (BPA)—used in the chemical industry have been considered as estrogenic EDCs. However, all of these EDCs have a very weak affinity for binding through the classical nuclear estrogen receptors (ERs), 1,000–2,000 times lower than that of E_2_ (Bonefeld-Jorgensen et al. 2001; Crain et al. 1998; Massaad and Barouki 1999). Studies of the nuclear transcriptional regulatory activities of nonphysiologic estrogens have mostly been unable to explain the actions of these chemicals in mediating endocrine disruption in animals and humans at the low picomolar or nano molar concentrations widespread in the environment (Calafat et al. 2005; Vandenberg et al. 2007). In the last few years, EDCs have been reported to act through hormone-independent mechanisms (Welshons et al. 2006) or through a nongenomic activation of membrane-initiated signaling pathways via membrane forms of ERs (Alonso-Magdalena et al. 2005; Bulayeva and Watson 2004; Nadal et al. 2000; Noguchi et al. 2002). Indeed, there is now convincing evidence that estrogens, in addition to the classical regulation of estrogen-responsive genes via nuclear ERs, are able to trigger rapid membrane activation of a variety of second-messenger–mediated signal transduction pathways (Kelly and Levin 2001; Vasudevan and Pfaff 2007), with possible implications for cell proliferation, apoptosis, or survival (Levin 2002). However, the nature of these membrane ER(s), their relation to the classical ERs, and the precise signaling pathways that are activated remain to be elucidated (Manavathi and Kumar 2006; Vasudevan and Pfaff 2007). Moreover, fetal exposure to xenoestrogens is believed to be involved in male reproductive and developmental patho genesis. Diethylestylbestrol (DES), a potent synthetic estrogen used as an antiabortive drug in the 1970s, has a well-known deleterious effect in adults exposed *in utero* (Newbold et al. 2006). DES can produce different developmental or carcinogenic effects in rodents (Newbold 2004) when given during specific developmental windows, including fetal or perinatal periods, such as cryptorchidism or breast, prostate, or endometrial cancers. However, although indirect epidemiologic data show a constant increase of testicular cancer in young men (Huyghe et al. 2007) and an increased relative risk via professional exposure to persistent organic pollutants, no experimental model has validated the possible carcinogenic role of exposure to xenoestrogens in developing a testicular germ cell cancer (Rajpert-De Meyts 2006).

BPA, initially produced like DES as a synthetic estrogen (Dodds and Lawson 1936), has been rapidly and widely used as a cross-linking chemical in the manufacture of polycarbonate plastic and epoxy resins. Because of incomplete polymerization and degradation of the polymers by exposure to higher than usual temperatures, BPA leaches out from food and beverage containers (Biles et al. 1997; Krishnan et al. 1993; Le et al. 2008), as well as from dental sealants. BPA is found in the serum, milk, saliva, and urine of humans at nanomolar concentrations (Calafat et al. 2005; Olea et al. 1996; Sun et al. 2004; Vandenberg et al. 2007). Remarkably, BPA has been measured in amniotic fluid at concentrations 5-fold higher than those measured in maternal plasma (Ikezuki et al. 2002). Fetal and perinatal exposures to BPA in rodents have been shown to affect the brain, mammary gland, and reproductive tract, including hormone-dependent cancer (Durando et al. 2007; Ho et al. 2006; Maffini et al. 2006; Markey et al. 2001; Munoz-de-Toro et al. 2005). Although BPA induces an estrogenic effect through classical nuclear ERs at high concentrations and with a reduced affinity relative to E_2_ (Gaido et al. 1997; Krishnan et al. 1993; Perez et al. 1998), it also is able to trigger a non genomic effect in pancreatic islet, endothelial, and hypophysial cells and in breast cancer cells by initiating rapid responses at low concentrations (Alonso-Magdalena et al. 2005; Bulayeva and Watson 2004; Nadal et al. 2000; Noguchi et al. 2002).

We recently reported that E_2_ coupled to bovine serum albumin (E_2_-BSA) stimulated the proliferation of human seminoma cells (JKT-1) *in vitro* through a G-protein–coupled nonclassical membrane ER (GPCR) (Bouskine et al. 2008). In the present study, we investigated the hypothesis that BPA could stimulate seminoma cell proliferation through such a nongenomic action. We observed a promoting effect of BPA on seminoma cells through a rapid activation of cAMP-dependent protein kinase (PKA) and cGMP-dependent protein kinase (PKG) signaling pathways via a GPCR, illustrating that xenoestrogens, suspected to act as deleterious factors in breast and prostate cancers, could also act in this nongenomic pathway as possible promoting agents in testicular germ cell cancer.

## Materials and Methods

### Cell culture and cell proliferation assay

JKT-1, a human testicular pure seminoma cell line developed from the testis of a 40-year-old man (Kinugawa et al. 1998), expresses placental alkaline phosphatase (PLAP), a classical seminoma marker (Roger et al. 2004) and more recently described markers (Santagata et al. 2007). Specific embryonic stem cell markers for the JKT-1 cell line have been described previously (Bouskine et al. 2008). JKT-1 cells were maintained in Dulbecco’s modified Eagle medium (DMEM; Gibco BRL, Cergy Pontoise, France) supplemented with penicillin/streptomycin (1%), sodium pyruvate (2%), and 10% fetal bovine serum (FBS) in a humidified 5% CO_2_ atmosphere at 37°C. Cells were seeded in six-well plates (0.6 × 10^6^ cells/well). After 48 hr, the cells were washed and estrogen-starved overnight in phenol red–free DMEM (Sigma, Lyon, France) supplemented with 1% charcoal-stripped FBS. We then added E_2_ (Sigma), freshly prepared E_2_-BSA (Sigma) devoid of free E_2_ removed by filtration (Bouskine et al. 2008), ICI 182780 (ICI; Falsodex; Astra-Zeneca, Birmingham, UK), BPA, DES, dichlorodiphenyltrichloroethane (DDT), DMSO (Sigma), or ethanol (as vehicle control) at different concentrations (picomolar to micromolar) according to previous studies (Bouskine et al. 2008; Roger et al. 2004) and at environmentally relevant BPA concentrations (Vandenberg et al. 2007) and incubated them for 24 hr.

We harvested cells using trypsin and counted them using Vi-CELL software (Beckman Coulter, Margency, France). Results are expressed as the percent of variation compared with control.

### Western blot analysis

We grew JKT-1 cells in 10-cm dishes at a density of 4.9 × 10^6^ cells per dish. After 48 hr, the cells were washed with phosphate-buffered saline (PBS) and incubated overnight in phenol red–free DMEM/0.1% BSA; cells were then exposed to ligands for different time periods. We purchased forskolin from Sigma and PD 98059 (PD), H89, KT5823, NF449, and pertussis toxin (PTX) from VWR Calbiochem (San Diego, CA, USA). After washing cells with PBS, we lysed cell pellets in ice-cold lysis Brij buffer/NP-40 [50 mM Tris-HCl (pH 7.5), 1% NP-40, 1% Brij 96 (Fluka, St. Quentin Fallavier, France), 1 mM Na_3_VO_4_, 10 mM β-glycerophosphate, 10 mM NaF, 2 mM EDTA, and protease inhibitors (Complete; Roche Diagnostics, Meylan, France)]. Lysates were sonicated 7 sec on ice twice and then centrifuged for 15 min at 14,000 rpm. Equal amounts of whole protein extract were resolved on a 9% SDS-polyacrylamide gel. Proteins were transferred to a polyvinyl difluoride Immobilon-P membrane (Millipore, Saint Quentin en Yvelines, France) and probed with the antibodies against phosphorylated cAMP response-element–binding protein (phospho-CREB; Cell Signaling, Boston, MA, USA) and phosphorylated retinoblastoma protein (phospho-Rb; BD Pharmingen, Le Pont de Claix, France). After the blots were stripped, we verified equal loading of proteins by reprobing the same blots with anti-actin antibody (Cell Signaling).

### Statistical analysis

Results of cell count or densitometric analysis are expressed as percentages of variation compared with control. We used a nonparametric Mann–Whitney test for statistical analysis.

## Results

### Low doses of BPA stimulate JKT-1 cell proliferation

**Figure 1:**
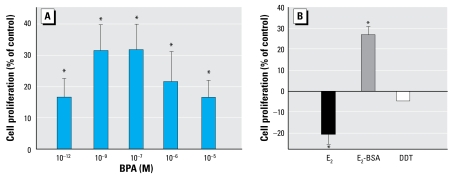
Stimulation of JKT-1 cell proliferation by 24-hr exposure to (*A*) various doses of BPA or (*B* ) E_2_ (10^−9^ M), E_2_-BSA (10^−9^ M), or DDT (10^−9^ M). Values shown are the percent change in cell number compared with control (steroid-free medium containing DMSO for BPA or medium containing ethanol for estrogens) given as the mean ± SE of three independent experiments. *p < 0.05.

BPA stimulated JKT-1 cell proliferation at very low doses. The dose–response curve had an inversed U-shape, with a weak stimulation at 10^−6^ M that was still present at 10^−12^ M, and a maximum effect around 10^−7^/10^−9^ M (Figure 1A). This stimulation was reproduced by E_2_-BSA, an impermeable E_2_ conjugate (Figure 1B), but not by E_2_, which triggered a significant decrease of cell proliferation at a physiologic intratesticular concentration of 10^−9^ M (Figure 1B), in agreement with our previous study (Roger et al. 2005).

**Figure 2:**
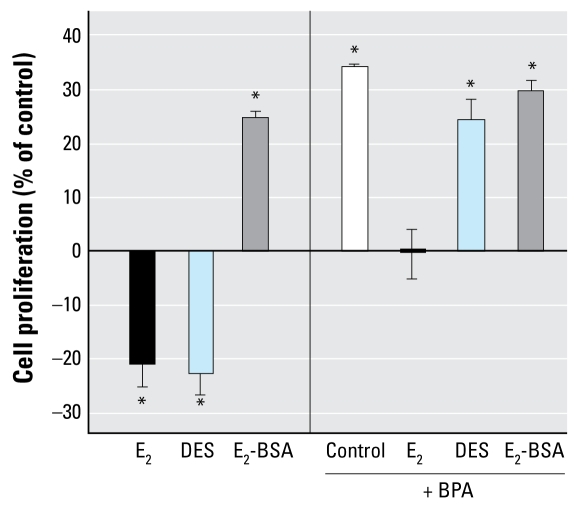
Effects of 24-hr exposure to E_2_, E_2_-BSA, or DES alone (each at 10^−9^ M) or combined with BPA (10^−9^ M) on cell proliferation in JKT-1 cells. Values shown are percent change in cell number compared with control (steroid-free medium containing DMSO for bisphenol A or medium containing ethanol for estrogens and DDT) given as the mean ± SE of three independent experiments. *p < 0.05.

DDT, an organochloride pesticide with antiandrogenic activity, had no effect on JKT-1 cell proliferation (Figure 1B). DES, a potent synthetic estrogen, acted as a suppressor in a dose-dependent manner, as did E_2_ (Figure 2). When BPA was combined with either E_2_ or DES to verify synergic or antagonist effect, BPA neutralized the suppressive effect of E_2_ (Figure 2) and completely prevented the DES suppressive effect at the dose tested (10^−9^ M). However, E_2_-BSA plus BPA induced the same effect of each alone, indicating a lack of synergic or antagonistic effect and likely a similarity in the activated pathways (Figure 2).

### BPA activates PKA and CREB in JKT-1 cells

**Figure 3:**
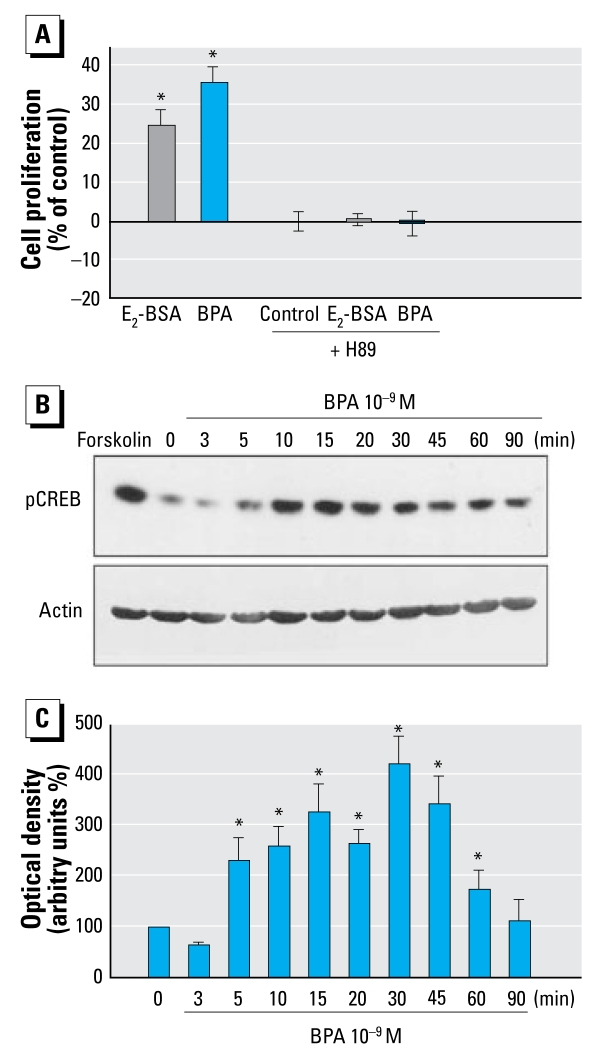
Promotion of cell proliferation in JKT-1 cells through activation of the PKA pathway. (*A*) Exposure to BPA or E_2_-BSA (both at 10^−9^ M) with or without inhibition of PKA by H89 (5 × 10^−6^ M) for 90 min; values shown are the percent change in cell number (mean ± SE) compared with control (steroid-free medium with DMSO for BPA or medium containing ethanol for E_2_-BSA). (*B*) Western blot analysis of CREB phosphorylation (pCREB; top) and actin expression in each sample (bottom) during exposure to 10^−9^ M BPA. Forskolin (25 μM) was used as a positive control for CREB activation. (*C*) Bands from three experiments were quantified by densitometry, and results were normalized to actin expression in each sample, plotted with SE, and compared with control (time 0). *p < 0.05.

Activation of PKA was necessary for BPA to promote JKT-1 cell proliferation because H89, a specific PKA inhibitor, totally prevented an increase in cell proliferation (Figure 3A). Extracellular stimuli elicit changes in gene expression in target cells by activating intracellular protein kinase cascades that phosphorylate transcription factors within the nucleus. CREB is one of these factors that activates gene transcription after the phosphorylation of serine 133 induced by a variety of protein kinases, including PKA and extracellular-signal-regulated kinase 1/2 (ERK1/2) (Shaywitz and Greenberg 1999). Using an anti–phospho-CREB antibody that recognizes phosphorylated serine 133, we observed a very rapid (5 min), BPA-induced activation of CREB in JKT-1 cells, with maximum activation at 30 min (Figure 3B). This activation was PKA dependent because H89 completely abolished CREB phosphorylation (data not shown).

### The cell cycle regulator Rb is phosphorylated during BPA-induced promotion of JKT-1 cells

**Figure 4:**
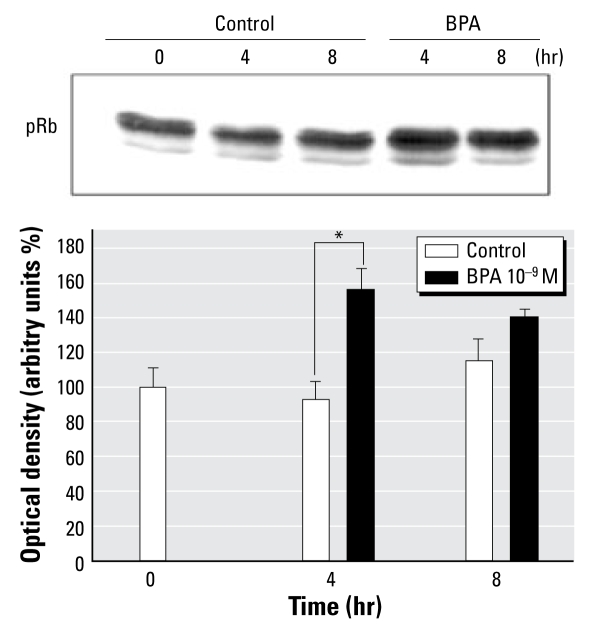
Activation of Rb phosphorylation during BPA activation. (*A*) Western blot analysis of Rb phosphorylation (pRb) during activation with BPA (10^−9^ M) for the indicated time points. (*B*) Bands from three experiments quantified by densitometry and compared with control (steroid-free medium containing ethanol). *p < 0.05.

Rb is a nuclear factor that participates in the regulation of the cell cycle, interfering with cyclin action when nonphosphorylated. This suppressive effect is prevented through phosphorylation of Rb. In JKT-1 cells, BPA induced a rapid (4 hr) and intensive phosphorylation of Rb (Figure 4), leading to Rb inactivation during BPA stimulation.

### ICI does not prevent BPA-induced JKT-1 proliferation

**Figure 5:**
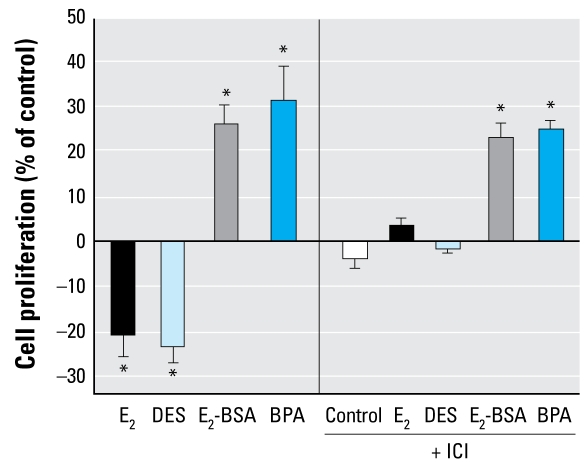
Effect of 90-min pretreatment with ICI on cell proliferation in JKT-1 cells treated for 24 hr with E_2_, DES, E_2_-BSA, BPA (all at 10^−9^ M). Values shown are percent change in cell number compared with control (steroid-free medium containing DMSO for BPA or medium containing ethanol for estrogens) given as the mean ± SE of three independent experiments. *p < 0.05.

We recently reported that JKT-1 cells express ER-β but not ER-α (Roger et al. 2005). By immunofluorescence, subcellular fractionation, and Western blot, we found that the ER-β receptor had an apparent intra-cytoplasmic localization without any evident membrane location (Bouskine et al. 2008). To determine whether ER-β was involved in the estrogenic or xenoestrogenic activation of JKT-1 cell proliferation, we tested the effect of ICI, a pure ER antagonist. ICI completely counteracted the suppressive effects of E_2_ and DES on JKT-1 cell proliferation (Figure 5), supporting an ER-β–dependent mechanism. However, ICI did not prevent the promoting effect of BPA or of E_2_-BSA, supporting our hypothesis that the rapid effect induced by these two ligands was likely not dependent on a classical ER (Figure 5).

### BPA stimulates proliferation in JKT-1 cells through a G-protein–coupled receptor (GPCR)

**Figure 6:**
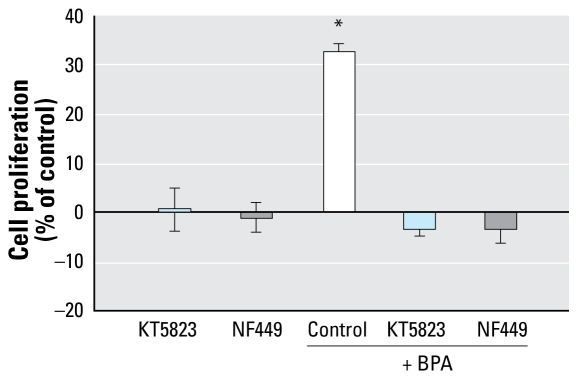
Effect of 90-min pretreatment with KT5823 (10^−6^ M) or NF449 (10^−5^ M) on BPA promotion of JKT-1 cell proliferation via a GPCR that involves both Gα_s_ and Gα_i_/Gα_q_ subunits. Cells were exposed to 10^−9^ M BPA for 24 hr. Values shown are percent change in cell number compared with control (steroid-free medium with DMSO) given as the mean ± SE of three independent experiments. *p < 0.05.

**Figure 7:**
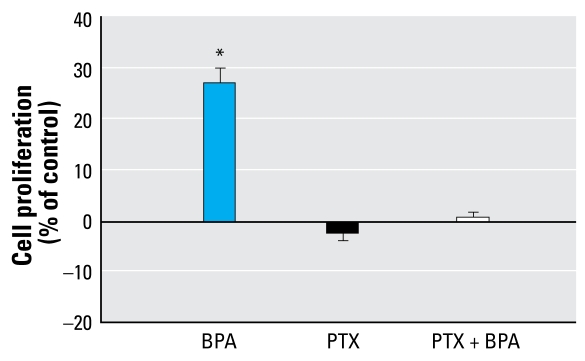
Effect of 90-min pretreatment with PTX (100 ng/mL) on GPCR-mediated activation of JKT-1 cell proliferation induced by 24-hr exposure to BPA (10^−9^ M). Values shown are percent change in cell number compared with control (steroid-free medium with DMSO) given as the mean ± SE of three independent experiments. *p < 0.05.

GPCRs have been proposed to be involved in triggering membrane action of steroids (Thomas et al. 2006), including estrogens (Filardo et al. 2002; Kelly and Wagner 1999). PKA activation is usually a Gα_s_ protein-dependent mechanism, so we tested the effect of NF449, a Gα_s_ inhibitor (Figure 6). NF449 blocked the promoting effect of BPA, illustrating the Gα_s_ dependence of the PKA activation and the G-protein–coupled nature of the receptor involved in BPA stimulation. We have previously shown that E_2_-BSA, which triggers an effect quite similar to that of BPA, also induces a Gα_i_-dependent activation of the mitogen-activated protein kinase (MAPK)/ERK1/2 pathway (Bouskine et al. 2008). For this reason, we also studied the effect of PTX, an inhibitor of Gα_i_/Gα_q_ protein, during BPA-induced JKT-1 proliferation. This toxin prevented the BPA-induced increase of cell proliferation (Figure 7).

### PKG pathway but not MAPK pathway is activated by BPA in JKT-1 cells

**Figure 8:**
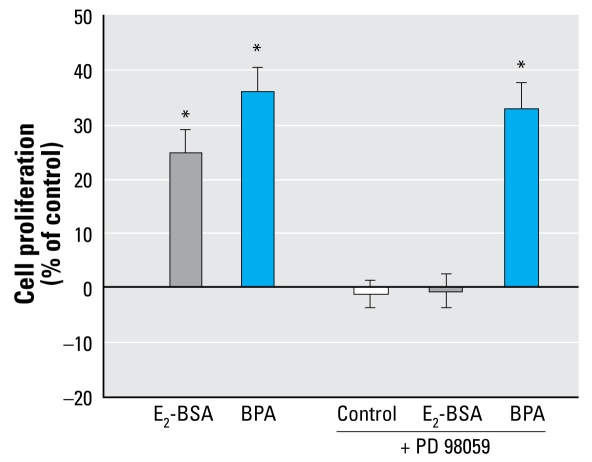
BPA promotion of JKT-1 cell proliferation is not ERK1/2 dependent. After 90-min pretreatment with PD (10^−6^ M), cells were exposed to E_2_-BSA or BPA (10^−9^ M) for 24 hr. Values shown are percent change in cell number compared with control (steroid-free medium containing DMSO for BPA and medium containing ethanol for E_2_-BSA) given as the mean ± SE of three independent experiments. *p < 0.05.

BPA promotion of JKT-1 cells did not seem to involve ERK1/2 activation because PD, an inhibitor of MAPK kinase, did not prevent BPA-enhanced proliferation (Figure 8). We therefore tested the PKG pathway, which is known to be Gα_i_/Gα_q_ dependent and is involved in BPA activation of calcium influx in pancreatic islet α cells (Alonso-Magdalena et al. 2005). KT5823, an inhibitor of PKG activation, prevented BPA-induced JKT-1 proliferation (Figure 6).

**Figure 9:**
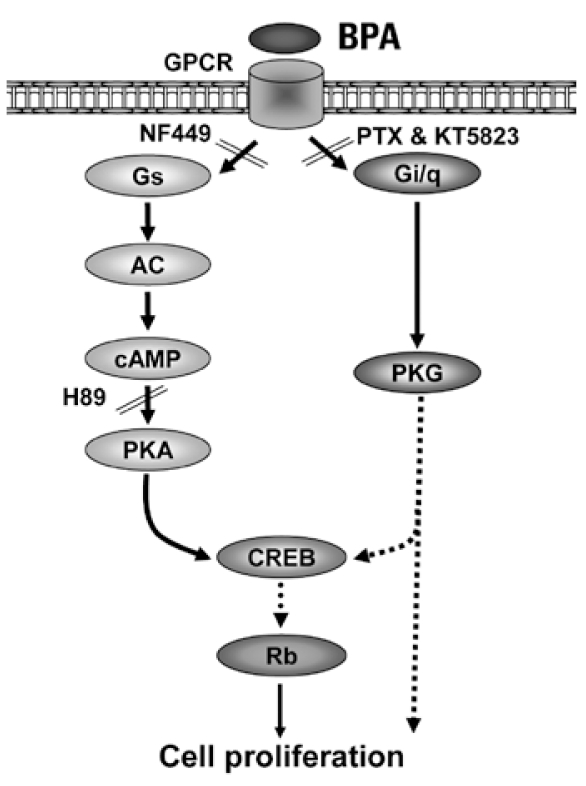
Proposed model showing the process by which BPA stimulates JKT-1 cell proliferation by activating PKA and PKG pathways via a nonclassical GPCR. Solid lines represent demonstrated steps, and dotted lines represent possible pathways yet to be demonstrated. AC, adenylate cyclase.

These results strongly support the participation of a membrane GPCR involving both the Gα_s_ and Gα_i_ subunits. Figure 9 summarizes the signaling pathways activated during BPA induced JKT-1 proliferation.

## Discussion

In this article we demonstrate for the first time that very low doses of BPA (picomolar or nanomolar) stimulate human seminoma cell proliferation by allowing a rapid, nongenomic, membrane-initiated activation of PKA and PKG signaling pathways associated with phosphorylation of the transcription factor CREB and the cell cycle regulator Rb. This promoting effect, similar to the one observed with E_2_-BSA but not with E_2_ alone (Bouskine et al. 2008), was triggered independently of classical ERs through a membrane receptor belonging to the GPCR family. The low concentrations of BPA able to produce such an effect give this observation environmental relevance and support the hypothesis of a possible contribution of xenoestrogenic fetal exposure to testicular germ cell carcinogenesis.

Estrogens classically mediate their action after binding to nuclear receptors that act as transcription factors to modulate the activity of target genes by interacting with several DNA response elements. In addition to their ability to mediate gene transcription, estrogens also elicit rapid nontranscriptional effects by membrane-mediated signaling pathways leading to calcium influx (Chaban et al. 2004), cAMP (Abraham et al. 2003) or nitric oxide production, phospholipase C activation, or inositol phosphate generation (Le Mellay et al. 1997). The MAPK/ERK1/2 pathway can also be rapidly activated by estrogens in various cell types, such as endothelial (Chen et al. 2004), adipocyte (Dos Santos et al. 2002), neuroblastoma (Watters et al. 1997), or breast cancer cell lines (Migliaccio et al. 1996). Membrane activation of these rapid signaling cascades will then modulate gene transcription (Vasudevan and Pfaff 2007). We have recently reported that human seminoma cells express both classical ER-β (Roger et al. 2005) and a membrane non classical estrogen GPCR (Bouskine et al. 2008). E_2_ has a high affinity for ER-β and triggers a suppressive effect in JKT-1 cells, whereas E_2_ coupled to BSA, which prevents membrane crossing, binds to an ncmER and promotes cell proliferation by activating rapid cell signaling, including PKA and MAPK pathways (Bouskine et al. 2008). BPA has a low affinity for ER-β, as described in several models, with a 1,000-fold weaker affinity than E_2_, and activates pancreatic islet, hypophysial, or endometrial cells through an ncmER (Alonso-Magdalena et al. 2005; Bulayeva and Watson 2004; Nadal et al. 2000; Noguchi et al. 2002). In JKT-1 cells, the differential affinity toward both receptors may explain the dose–response curve observed. At high micromolar concentrations, BPA may trigger a suppressive effect via ER-β as does E_2_, which neutralizes the nongenomic effect. At low concentration (10^−9^ M), this genomic effect is absent, allowing the non-genomic effect to be displayed because of the high affinity of BPA for the ncmER. When mixed together at this low concentration, BPA and E_2_ are mutually antagonistic, whereas DES, also a potent ligand for nuclear ER, at this low concentration only moderately counteracts the nongenomic BPA effect, possibly for conformational reason. Our model illustrates the paradoxical inversed U-shaped curve, explaining effects at very low doses (Brucker-Davis et al. 2001), that has been described for BPA in several models (Maffini et al. 2006; vom Saal and Hughes 2005; Welshons et al. 2006), which could be produced by two different ERs and two different, genomic and non-genomic, mechanisms.

We propose that the promoting effect occurs through nongenomic transduced activation of the PKA/CREB and the PKG pathways, as illustrated by the very rapid phosphorylation of CREB and the inhibition of both CREB phosphorylation and proliferation obtained with the PKA antagonist H89 and the PKG inhibitor NF449. Phosphorylated CREB will regulate cell-cycle–controlling genes as demonstrated by Rb phosphorylation. Estrogenic activation of the PKA/CREB pathway through an ncmER has already been described in several models (Belcher et al. 2005; Filardo et al. 2002; Quesada et al. 2002). Concerning ERK activation, Wozniak et al. (2005) assessed the rapid changes in intracellular calcium levels induced by xenoestrogens in a pituitary tumor cell line and found that multiple membrane-initiated signaling pathways were activated. The differential patterns presented seemed to depend on the structure of the xenoestrogens and the conformation obtained with the membrane ER (Wozniak et al. 2005). In particular, BPA was one of the EDCs tested that did not induce ERK activation (Wozniak et al. 2005), as in our seminoma cell model. In another model of pancreatic islet α cells, Alonso-Magdalena et al. (2005) showed that estrogens induce a rapid calcium influx through several pathways, including PKG. We therefore tested the PKG pathway, which involves for its activation a Gα_q_ subunit, and found its contribution in BPA-induced JKT-1 proliferation as well, likely through a rapid intra cytoplasmic calcium increase. Then, we showed that the BPA proliferation-promoting effect on semi-noma cells in fact needed the two subunits Gα_s_ and Gα_i_, which acted not in an opposite but in a complementary fashion, as already described in other models (Daaka et al. 1997) and supported in JKT-1 cells by the use of specific inhibitors. Indeed, activation of both the PKA and PKG pathways seemed to be necessary for the BPA-induced promoting effect, as we have previously shown for E_2_-BSA with the two PKA and ERK pathways (Bouskine et al. 2008). BPA, when mixed with E_2_-BSA, showed the same proliferative effect as each compound alone, without any synergistic or antagonistic effect. Despite the mild difference in the activated protein kinases, it is likely that this promoting effect induced by E_2_-BSA and BPA is mediated via the same GPCR membrane receptor, an ncmER.

Increasing evidence from different tissues and cell types has suggested that there are multiple mechanisms through which estradiol can stimulate rapid intracellular signaling (Kelly and Levin 2001; Manavathi and Kumar 2006). However, one of the main questions remains the nature of the coupled receptors. Different studies of nonclassical estrogen signaling in a variety of target cells, such as endothelial, neuronal, and pituitary cells (Kim et al. 1999; Li et al. 2003), have strongly suggested that nuclear classical ER or ER-like proteins are candidates (Pedram et al. 2006) for the membrane ERs. In our model, however, this membrane receptor is unlikely to be a classical ER because ICI failed to inhibit cell proliferation; JKT-1 cell membranes do not express ERβ as we previously reported (Bouskine et al. 2008); and E_2_ and DES, a potent synthetic estrogen that binds to ER, do trigger not a promoting but a suppressive effect. Filardo et al. (2002) showed that estrogen-induced ERK activation may occur in human breast cancer cells that do not express either ER-α or ER-β. GPR30, an orphan GPCR, has been proposed as a nonclassical estrogen receptor able to stimulate cancer cells (Albanito et al. 2007; Filardo 2002; Revankar et al. 2005; Thomas et al. 2005; Vivacqua et al. 2006). Thus, GPR30 is a candidate for our ncmER in JKT-1 seminoma cells, activated by E_2_-BSA or BPA and able to transduce the PKA and ERK or PKG signaling pathways. Moreover, GPR30 has been identified recently in mouse spermatogonia and has also been involved in estrogenic germ cell proliferation control (Sirianni et al. 2008). Seminoma cells are considered to be issued from transformed gonocytes or undifferentiated spermatogonia (Rajpert-De Meyts 2006). Thus, we propose that GPR30 could represent the ncmER in JKT-1 seminoma cells, able to activate PKA, ERK, or PKG pathways. Studies of its expression, precise localization, and involvement in triggering an estrogenic promoting effect in human seminoma cells and human fetal or adult germ cells are now under way in our laboratory.

It is now possible, as we recently proposed (Bouskine et al. 2008), to more comprehensively describe actions of estrogens at low concentrations on human seminoma cells, involving two different and opposite effects: first, a predominant long-lasting, suppressive effect, antagonized by ICI, thus likely mediated by ER-β, which involves a classical nuclear genomic pathway controlling cell cycle gene expression (Roger et al. 2005); and second, a rapid, nongenomic promoting effect triggered by PKA and ERK activation, which involves not a classical ER (not antagonized by ICI or tamoxifen), but rather a PTX-responsive non-classical membrane estrogen GPCR (Bouskine et al. 2008). The resulting impact on germ cells may depend on the relative expression of both receptors (ER-β and GPCR), the endogenous concentration of E_2_, and the respective binding affinity of the estrogenic compounds. In the presence of both receptors, as in JKT-1 seminoma cells, the suppressive effect of E_2_ may remain predominant because of a rapid dephosphorylation of ERK1/2 by an ER-β-dependent expression of protein phosphatase 2A, as described in neonatal rat cerebellar neurons (Belcher et al. 2005). In contrast, in JKT-1 cells, BPA does not need the MAPK pathway, but rather the PKG pathway, which would have a more potent effect on the nongenomic pathway because BPA has a higher affinity for this receptor. Furthermore, human gonocytes, which do not express the active ER-β_1_ isoform until the prenatal period (Gaskell et al. 2003), may be exclusively sensitive to the membrane-mediated promoting effect if they do express the ncmER as do mouse spermatogonia (Sirianni et al. 2008). Progressive expression of ER-β_1_ during the perinatal period will prevent proliferation and enhance differentiation into spermatogonia. However, excessive fetal exposition to xenoestrogens with high affinity for the non classical estrogen GPCR, as shown here for BPA, may promote abnormal proliferation of gonocytes through the nongenomic pathway and thus contribute to malignant germ cell transformation, leading, as proposed by Skakkebaeck et al. (1998), to carcinoma *in situ* and then to testicular germ cell cancer, the most frequent cancer of the young men with an increasing incidence.

This GPCR-mediated nongenomic action therefore represents a new basis for evaluating xenoestrogens such as BPA that could interfere with the developmental programming of fetal germ cell proliferation and/or differentiation when it crosses the placenta. Testing of EDCs will require a cell model that expresses this receptor alone and/or together with nuclear ERs.
